# On the pivotal role of dose for particle toxicology and risk assessment: exposure is a poor surrogate for delivered dose

**DOI:** 10.1186/s12989-017-0233-1

**Published:** 2017-12-08

**Authors:** Otmar Schmid, Flemming R. Cassee

**Affiliations:** 1 Comprehensive Pneumology Center (CPC-M), Member of the German Center for Lung Research (DZL), Max-Lebsche-Platz 31, 81377 Munich, Germany; 2 Institute of Lung Biology and Disease, Helmholtz Zentrum München – German Research Center for Environmental Health, Ingolstaedter Landstrasse 1, 85764 Neuherberg, Germany; 3 National Institute for Public Health and the Environment (RIVM), PO box 1, 3720 BA Bilthoven, The Netherlands; 4 Institute for Risk Assessment Sciences, Utrecht University, Utrecht, The Netherlands

## Introduction

There is still concern regarding potential health effects of nano-sized and nano-structured materials (NMs). Since reliable risk assessment is essential for sustained societal acceptance, there has been substantial funding for toxicological studies in the past decades. Albeit a wealth of data has been published since, the relevance of those data for risk assessment can be questioned. This is particularily the case for data from studies that focused on inhalation as the route of exposure. The derivation of exposure limits for NMs is still an elusive issue, although some regulatory measures have been undertaken e.g. for inhalation of carbon nanotubes at the workplace [1].

Today, one of the main obstacles for risk assessment of nano- and micronsized particles and fibres is not the lack of toxicological data, but a shortage of data meeting all of the criteria for use in risk assessment. One of the most important criteria is accurate dosimetry. While most studies provide accurate data on particle exposure concentration, i.e. mass of particles per volume air (μg/m^3^) or – for in vitro submerged cell-based assays – per volume cell culture medium (μg/cm^3^), the pivotal role of dose delivered to the site of exposure (e.g. lung epithelium (in vivo) or lung cell culture (in vitro)) has been widely overlooked. This is especially the case in - but not limited to - studies with in vitro cell systems [2, 3].

In addition, the choice of consistent and relevant dose metrics (e.g. mass, volume, surface area, number) has also been identified as one of the key issues for limited applicability of toxicological data for risk assessment [4]. While this is beyond the scope of this editorial, we mention that current results indicate that – in addition to mass, the traditionally used metric – at least one more dose metric should be used such as surface area, volume or number, depending on the type of NM [5–7]. This editorial highlights the fundamental role of delivered particle dose (sometimes also referred to as internal dose) for translation of toxicological dose-response data into risk assessment and exposure limits. The concept of delivered dose is of overarching significance for any type of particle exposure scenario. For simplicity we are focusing on poorly soluble particles (PSP) and models for respiratory toxicity assessment, i.e. inhalation exposure for in vivo studies and cell culture exposure under submerged or air-liquid interface conditions.

## Exposure or dose: Two sides of the same “coin” in PSP risk assessment

**Fig. 1:**
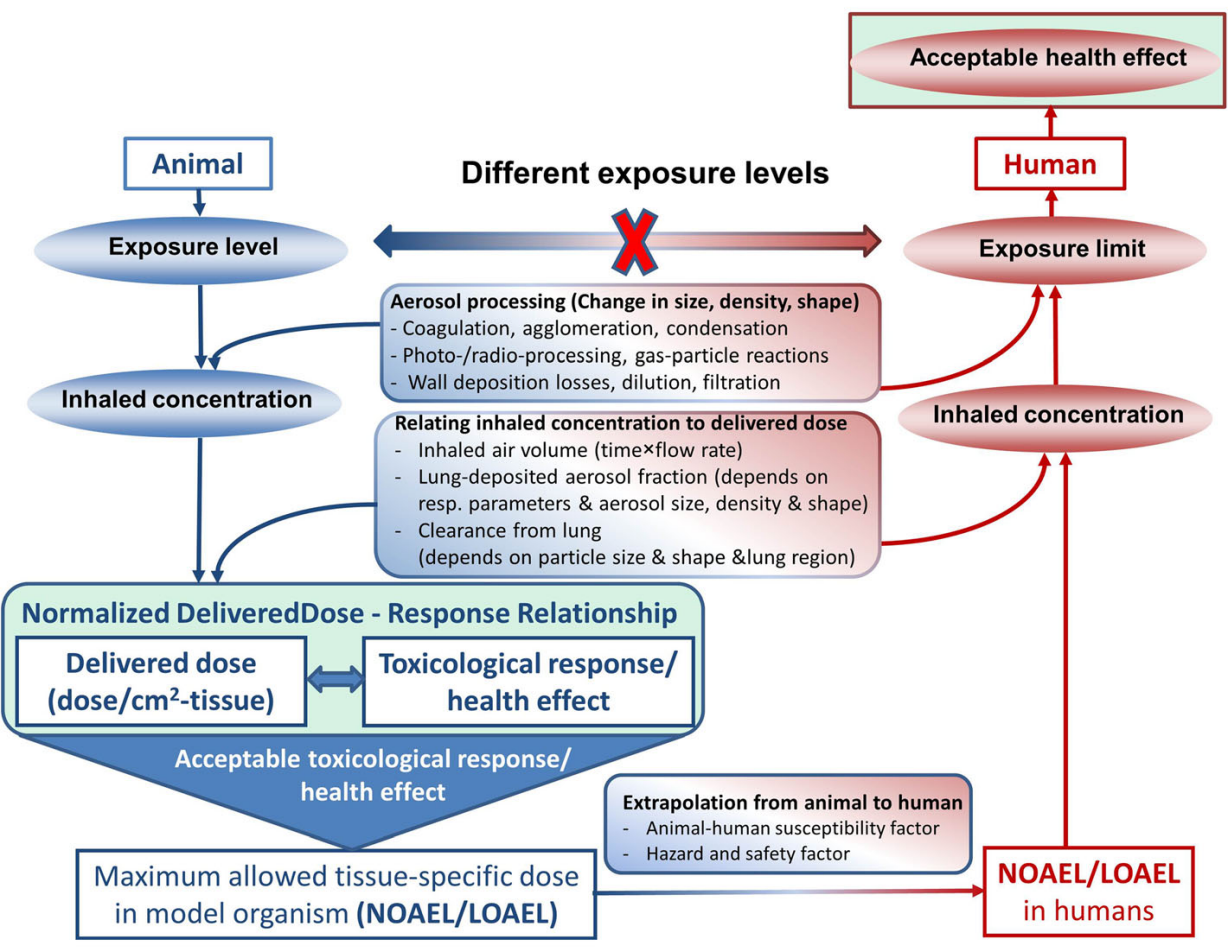
Normalized delivered dose (dose per surface area or mass of lung/tissue) and not exposure plays the pivotal role for derivation of human exposure limits from toxicological inhalation studies. The main factors influencing conversion of PSP aerosol exposure concentration into (lung-) delivered dose are depicted for animal inhalation experiments and factors relevant for extrapolation of No or Low Observed Adverse Effect Levels (NOAEL/LOAEL) from animal models to humans are highlighted

Often, exposure is considered an acceptable measure for dose in hazard and risk assessment studies. One of the reasons for this false perception lies in the different perspectives on risk taken by the regulatory body and the toxicological community. In contrast to exposure concentration, delivered dose is often difficult to measure and real-time dose measurement devices are often not available at all. For practical reasons, regulatory measures of health protection have to set limits based on readily measurable quantities of exposure, namely exposure concentration levels (“exposure limit”). Ideally, these exposure limits can be adjusted to any substance by determination of material-specific hazard factors. This concept of hazard-based risk assessment is often presented in a simplified form describing risk as a function of exposure and hazard (risk=f[exposure, hazard]). However, this is misleading, since exposure to an aerosol of PSP is a poor predictor of delivered dose (see Fig. 1) and from a toxicological point of view only the delivered dose, i.e. the dose which comes into contact with an organism, can do harm. For inhalation the dose of PSP is strongly dependent on many factors including respiratory parameters (tidal volume, breathing frequency) and PSP-lung deposition which depends on PSP characteristics affecting the diffusive (mobility) and aerodynamic behaviour of PSP (size, density and shape) [8]. Hence, the relevant measure for toxicological dose-response analysis with relevance for human risk assessment can only be *delivered dose* – not exposure. For definition of exposure limits, dose-based toxicological results have to be translated into equivalent exposure limits as described below.

## The role of delivered dose for risk assessment

As mentioned above reliable risk assessment of PSP hinges on (delivered) dose-response relationships mostly obtained from animal inhalation experiments. Derivation of dose-based Low or No Observed Adverse Effect Levels (LOAEL/NOAEL) and related human exposure limits require extrapolation of LOAEL/NOAEL from animal/cell to human conditions taking into account aerosol processing mechanisms and factors determining the relationship between inhaled concentration and delivered dose (see Fig. 1) [9–11]. Importantly, inter-species dose conversion (allometric scaling) requires appropriate normalization of the delivered dose. For inhaled PSP this is typically done based on mass of the lung or surface area of the lung epithelium (in vitro: surface area of cell culture system), i.e. ideally the *normalized delivered dose* should be presented in terms of μg/cm^2^-tissue (lung epithelium) or μg/g-lung (e.g. wet lung weight of mouse and rat vary around 0.18 and 1.3 g, respectively [10]. It is noteworthy, that allometric scaling is often based on body weight, but for inhaled substances surface area (or mass) of the lung is more relevant than body weight [7].

Due to practical reasons most animal inhalation studies report dose-response relationships and NOAEL/LOAEL in terms of retained dose, i.e. delivered dose corrected for PSP clearance from the lung due to mucociliary and macrophage activity [12]. To the best of our knowledge, it has not been investigated whether delivered or retained dose of PSP is more predictive for adverse health outcome. For humans and commonly used animal models the PSP clearance rates are well known and incorporated in computational dosimetry models (e.g. ICRP, MPPD) allowing for easy conversion from delivered to retained dose, if the exposure regiment (timing) and rate of delivered dose are known [13, 14]. Hence, at this time both delivered and retained dose are acceptable, but should be clearly identified and all parameters for conversion into each other should be documented.

As a consequence, derivation of human exposure limits from toxicological studies relies on dose-response data in terms of *normalized delivered dose*, not in terms of exposure. In other words, exposure-response curves in animal or cell models do not allow for direct conclusions on human exposure limits, i.e. for any toxicologically equivalent *normalized dose* the corresponding exposure levels in animals and humans are different (Fig. 1).

## The concept of delivered dose in in vitro studies

Historically, mainly in vivo animal studies were used for derivation of exposure limits and hazard factors, while in vitro cell models mainly contributed to identification and understanding of toxicological pathways. With the advent of more complex in vitro models of the lung and other organs, as well as from the perspective of the 3R principles of more ethical animal testing (calling for refinement, reduction and replacement of animal testing), in the future a more significant role of in vitro testing for risk assessment of particles and nanomaterials can be expected.

In principle, two types of in vitro particle exposure systems can be distinguished. Either aerosolized particles are deposited directly onto cell models of the lung cultured at the air-liquid interface (ALI) or particles are suspended in cell culture medium and incubated with cells under submerged culture conditions, i.e. cells are completely covered with particle-laden cell culture medium. Clearly, ALI exposures are mimicking the physiologic conditions in the lung more realistically and are therefore potentially more predictive for inhaled particles [12, 15]. Moreover, under ALI conditions the *(normalized) delivered dose* can be either determined directly using e.g. quartz crystal microbalances (QCMs) [16] or predicted by computational or semi-empirical aerosol deposition models taking into account all relevant aerosol properties and the experimental characteristics of each exposure system [13, 14]. On the other hand, exposures under submerged conditions are relatively easy to perform by just adding a known amount of PSP to cell culture medium. However, this is deceiving, since a substantial amount of effort has to go into preparation of stable particle suspensions and – even more problematic – into determination of the fraction of the administered dose (dose added to the medium) which is finally delivered to the cells [17, 18]. In the past, the preference of delivered dose over administered dose has been largely ignored or overlooked and only recently practical tools for determination of the (normalized) delivered dose in submerged cell systems have been presented [19, 20]. However, validation of these dosimetry models is hampered by uncertainty if cellular uptake of PSP is required for toxicological response or if contact between the cell membrane and PSP may be enough for stipulating toxicological response. Nevertheless, particokinetics models or cellular uptake/delivery measurements are currently recommended for estimation of the delivered dose.

**Fig. 2:**
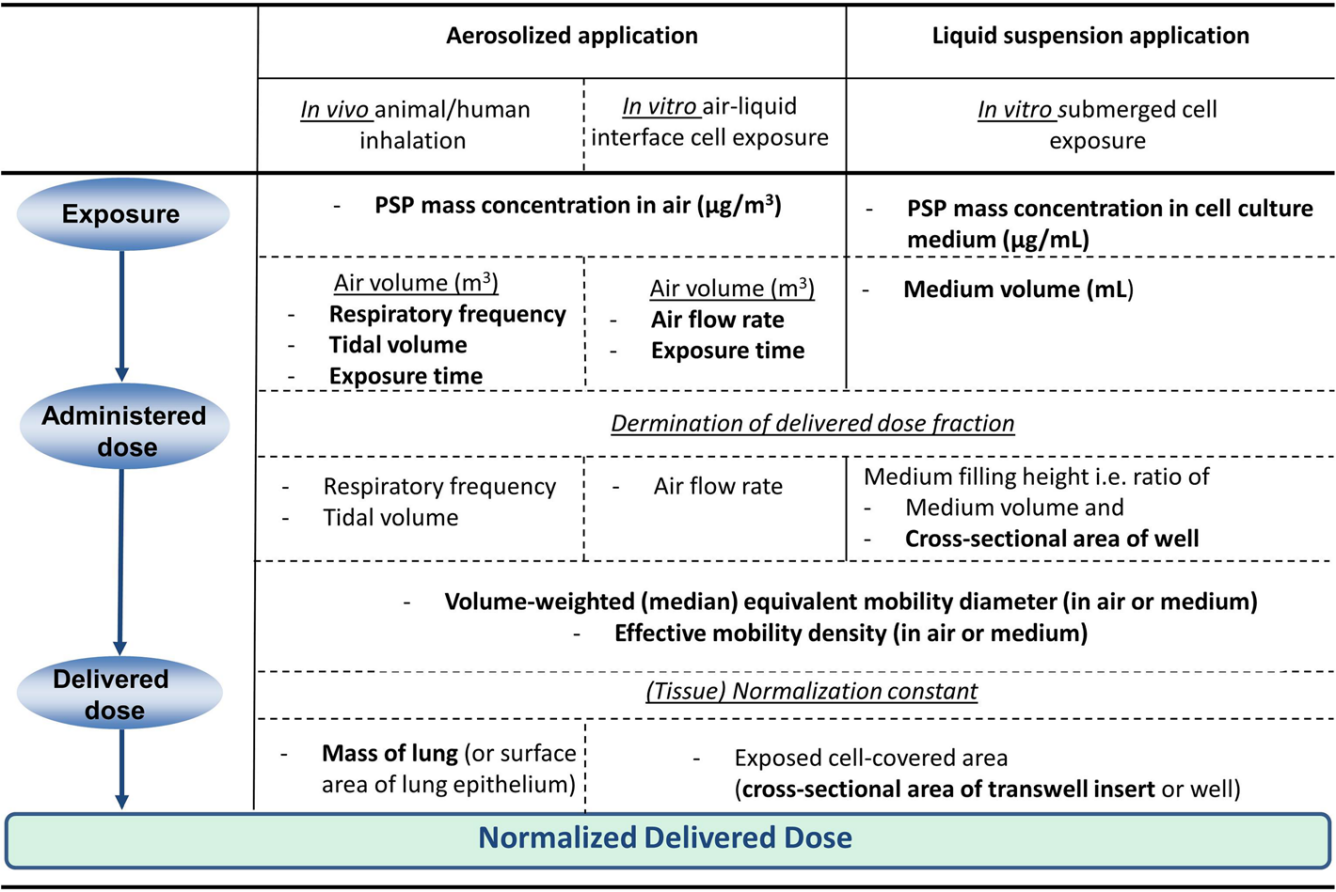
Required sets of experimental parameters for conversion of poorly soluble particle (PSP) exposure concentration into normalized delivered dose using particle-lung deposition and particokinetics models for in vivo inhalation (e.g. MPPD V3.04, [13]) and in vitro exposures under air-liquid interface [9] or submerged exposure conditions (ISDD model, [14, 19]). The list of required parameters (without repetitions) is given in bold letters for each of the three exposure scenarios. As caveat we add that toxicological measurements should be performed under stable particokinetic conditions, i.e. both mobility diameter and effective mobility density should be constant during the exposure time

To avoid uncertainties with particokinetics models, the delivered dose should be measured directly. In principle this is easily possible for in vitro air-liquid interface cell exposure experiments (e.g. QCM) and somewhat more complicated for in vivo animal and submerged in vitro cell experiments, but material-specific limitations may apply e.g. for carbonaceous PSP, which are difficult to detect in (carbon-dominated) biological matrices. Alternatively, the delivered dose can be inferred from the exposure concentration and particle deposition models, if a set of five to seven parameters is known, which depends on the type of exposure scenario as presented in Fig. 2. In all of these scenarios, the exposure concentration is multiplied by the volume of air (or cell culture medium) to calculate the administered dose which is subsequently converted into delivered dose utilizing particle-lung deposition models for animal inhalation experiments or particle-cell deposition (particokinetics) models for in vitro studies with air-liquid interface or submerged cell culture systems. For all of these three exposure scenarios the main PSP deposition mechanisms are typically diffusion, sedimentation, interception (relevant for high aspect ratio PSP) and/or impaction as well as electrophoresis and thermophoresis for some air-liquid interface cell exposure systems [12, 21]. Any of these processes can be described using a set of parameters related to either the exposure system or PSP characteristics, namely particle size, shape and density (plus electrical net charge for electrophoresis). It has been shown that these three PSP parameters can be lumped into two directly measurable parameters referred to as volume-weighted (median) equivalent mobility diameter and effective mobility density (ratio of PSP mass and equivalent mobility volume (= π/6 *d*_*m*_^3^, with *d*_*m*_ = equivalent mobility diameter) [6]. For easy and reliable particokinetics modelling, both equivalent mobility diameter and effective mobility density should be stable during the entire exposure period. As mentioned above, in addition to these particle characteristics, three to five exposure-related experimental parameters have to be reported depending on the exposure setup as specified in Fig. 2.

## Conclusions and recommendations

Delivered dose – not exposure concentration - is absolutely crucial for consistency, risk assessment and regulatory relevance of inhalation particle toxicological studies, since it allows for inter-comparability between in vivo and in vitro data, read-across analysis (prediction of effects of the same (or similar) chemical entity with varying physical characteristics), and extrapolation of cell or animal data to human exposure conditions and subsequent conversion to exposure limits.

From this conclusion the following recommendations can be derived for studies on inhalation toxicology of poorly soluble particles (PSP):

- Cell/lung delivered PSP dose should be determined and normalization of dose to cell/lung surface area is essential for extrapolation of dose levels from cell culture or animal models to humans.
- In addition to mass, which is still the most relevant metric for regulatory purposes, a biologically more relevant dose metric should be chosen. For non-fibre-specific toxicity, BET surface area seems to emerge as the most promising metric, but no final recommendation can be made at this time.
- If only concentration instead of delivered dose can be provided, sufficient physical characterisation of the PSP and the experimental conditions has to be provided to allow for conversion from concentration to dose (see Fig. 2).
- Likewise, for in vitro PSP exposures with submerged cell culture systems normalized delivered dose is the relevant measure for extrapolation to humans. Hence, either the delivered dose shoud be determined experimentally or the following parameters should be reported for submerged cell culture studies:
  ○ All relevant experimental parameters for conversion of particle concentration (μg/ml-medium) into administered dose (μg/cm^2^-cells) should be reported, namely exposure concentration in cell culture medium and medium filling height (derived from cell covered area and medium volume per well) (Fig. 2).
  ○ State-of-the-art methods should be used for conversion of administered to delivered dose and all relevant experimental parameters for conversion of administered to delivered dose should be reported [18]. Currently, the most widely used set of parameters is volume-weighted equivalent mobility diameter and effective mobility density (ratio of mass and mobility-diameter-based volume assuming spherical particle shape) (Fig. 2).
